# Taurine Mitigates Metaflumizone‐Induced Hepatonephrotoxicity in Rats by Inhibiting Oxidative Stress, Inflammation, and Apoptosis

**DOI:** 10.1002/jbt.70335

**Published:** 2025-06-09

**Authors:** Hasan Huseyin Demirel, Sinan Ince, Fahriye Zemheri‐Navruz, Sevim Feyza Erdogmus, Nilay Isitez

**Affiliations:** ^1^ Bayat Vocational School Afyon Kocatepe University Afyonkarahisar Türkiye; ^2^ Faculty of Veterinary Medicine, Department of Pharmacology and Toxicology Afyon Kocatepe University Afyonkarahisar Türkiye; ^3^ Faculty of Science, Department of Molecular Biology and Genetics Bartın University Bartın Türkiye; ^4^ Department of Basic Pharmaceutical Sciences, Faculty of Pharmacy Afyonkarahisar Health Sciences University Afyonkarahisar Türkiye

**Keywords:** apoptosis, hepatonephrotoxicity, inflammation, metaflumizone, oxidative stress, taurine

## Abstract

Metaflumizone (MTF) is a pyrazoline sodium channel blocker (SBI) insecticide, and data on its toxicity are limited. Taurine (2‐aminoethanesulfonic acid) is a sulfur‐containing β‐amino acid that is naturally found in high concentrations in cells. In this study, we thoroughly evaluated the impact of taurine on MTF‐induced hepatonephrotoxicity in a rat model, focusing on oxidative stress, inflammatory responses, and programmed cell death. In the present study, MTF (500 mg/kg, orally) to induce hepatonephrotoxicity was delivered to male rats for 30 days, and taurine at different concentrations (50, 100, and 200 mg/kg, orally) was used for protective effect for the same period. Taurine treatment alleviated the elevated levels of AST, ALT, ALP, BUN, and creatinine caused by MTF. It further suppressed malate dehydrogenase levels and enhanced antioxidant defense by elevating SOD, GSH, and CAT levels. Additionally, taurine increased the mRNA expression levels of *Bcl‐2*, which had been reduced due to oxidative stress, inflammatory, and apoptotic pathways, while suppressing the elevated gene expression levels of *NFκB*, *TNF‐α*, *Bax*, and *Cas‐3*. Furthermore, taurine regulated the altered protein expression levels of *Bcl‐2*, *Bax*, and *TNF‐α* induced by MTF. Microscopically, taurine also mitigated liver and kidney tissue damage caused by MTF. In conclusion, taurine significantly reduced MTF‐induced hepatonephrotoxicity by suppressing oxidative stress, inflammatory responses, and programmed cell death. These findings indicate that taurine has the potential to be a treatment option in the case of the prevention of liver and kidney damage caused by SBI.

## Graphical abstract

The taurine treatments in a dose‐dependent manner significantly reduced MTF‐induced hepatonephrotoxicity by suppressing oxidative stress, inflammatory responses, and apoptosis.

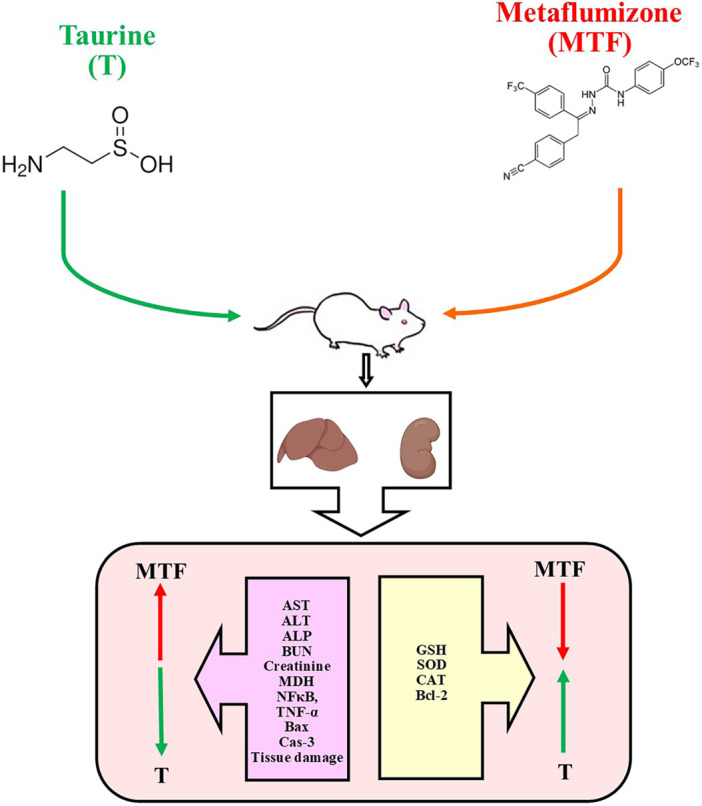

## 1 Introduction

Metaflumizone (MTF) belongs to the semicarbazone insecticide class and acts as a sodium channel blocker (SBI) in the nervous system of insects, leading to paralysis [1]. It has a low‐risk potential for nontarget organisms, including humans, and exhibits weak acute toxicity via oral, dermal, and inhalation exposure [2]. Additionally, MTF does not demonstrate mutagenic or genotoxic effects at normal application concentrations [3]. In cases of high‐dose exposure, it has been reported to induce methemoglobinemia in humans [4], and, in rats, oral administration at 300 mg/kg over a subchronic period resulted in reduced food consumption and mild liver lesions [3]. However, to date, there is no available information on the impact of MTF on oxidative stress and inflammation in mammals.

Taurine (2‐aminoethanesulfonic acid) is a member of the family of sulfur‐containing amino acids that includes methionine, cysteine, and homocysteine [5]. The majority of taurine absorption occurs in the gastrointestinal tract within 1–2.5 h after ingestion [6]. Plasma taurine returns to basal concentrations 6–8 h after ingestion, and the kidneys regulate its concentration through urinary excretion (between 65 and 250 mg per day) [7]. The daily intake of taurine from food alone is approximately 40‐400 mg/day, and clinical studies of taurine have shown that the amount without side effects is 14 g/day, which is considered the safe observed for normal healthy adults [8]. Unlike other sulfur‐containing amino acids such as methionine and cysteine, no comprehensive study provides a clear basis for toxicity for taurine. However, taurine intake has been reported to provide therapeutic benefits in the treatment of diabetes, hypertension, heart failure, retinal degeneration, and skeletal muscle disorders [9]. Additionally, the knowledge that taurine plays a role in osmoregulation, regulation of cellular calcium levels, and its high concentration in muscle tissue has led to its importance in the prevalence and research of energy and sports drinks containing it [6, 10]. Also, taurine mitigates oxidative stress‐induced toxicity in the liver and kidney, thereby preventing inflammation and apoptosis [11, 12, 13]. However, the potentially toxic effects of MTF on the liver and kidneys, as well as the protective role of taurine against these effects, have not yet been fully investigated. This study investigates novel findings from in vivo, the protective potential of taurine against MTF‐induced oxidative stress in rats. To accomplish this, liver and kidney function parameters and antioxidant status were biochemically assessed, along with histopathological evaluations. Furthermore, the expression levels of inflammatory cytokines and apoptotic markers at both mRNA and protein levels in kidney and liver tissues were examined.

## 2 Materials and Methods

### 2.1 Chemicals

For the study, MTF (CAS no: 139968‐49‐3) and taurine (CAS no: 107‐35‐7) were sourced from BASF (Istanbul, Turkey) and Sigma‐Aldrich (Missouri, USA), respectively. All additional chemicals utilized in the study were acquired from commercial vendors.

### 2.2 Animals and Experimental Protocol

Thirty male Sprague‐Dawley rats were acquired from the Experimental Animal Research and Application Unit at Afyon Kocatepe University. This study adhered to universal ethical guidelines, and approval was secured by the ethics committee (49533702/20). The rats were sustained in a standard surrounding environment, having a temperature of 22 ± 3°C, humidity levels of 50%–55%, and a 12‐h light/dark cycle. The subjects were randomly distributed into five distinct groups. Each consisted of six rats and was given a 7‐day acclimatization period. During the experiment, standard rodent chow and 1 mL of physiological saline were given orally to the control group. 500 mg/kg of MTF dissolved in physiological saline was given orally for 30 days to the MTF group. 50, 100, and 200 mg/kg of taurine dissolved in physiological saline were administered orally to the taurine and MTF groups for 30 days. The dose of MTF was determined based on preliminary trials, set at 1/10th of the acute oral lethal dose 50 in rats, where oxidative stress was observed. The doses of taurine were selected based on previous toxicological studies, in which taurine exhibited a significant protective effect [13, 14].

Twenty‐four hours after the final treatment, the rats were anesthetized through an intraperitoneal injection of ketamine (84 mg/kg) and xylazine (11.2 mg/kg). After 20 min, blood samples were collected via intracardiac puncture, and liver and kidney tissues were harvested. Plasma was isolated from the collected blood, and parts of the kidney and liver tissues were mixed thoroughly in 0.15 M Tris‐HCl buffer (pH 7.4) for biochemical analysis. For genetic studies, tissue specimens were immediately cryopreserved in nitrogen and stored at −80°C till further use. Histopathological specimens were fixed in 10% formaldehyde and subsequently prepared for examination.

### 2.3 Biochemical Parameters

The levels of alanine aminotransferase (ALT; LOT:200005), aspartate aminotransferase (AST; LOT:200003), alkaline phosphatase (ALP; LOT:190006), blood urea nitrogen (BUN; LOT:16015), and creatinine (LOT:101524B) in the plasma of the rats were assessed and quantified spectrophotometrically using commercially available kits from BIOLABO (Medica, Germany) with a Shimadzu 1601 UV‐VIS spectrophotometer (Tokyo, Japan).

### 2.4 Oxidative Stress and Antioxidant Enzyme Activities

The levels of malate dehydrogenase‐1 (MDH), which increase as a result of oxidative stress, were assessed in plasma and tissues using a double antibody‐Sandwich ELISA kit (FineTest, Cat No: ER1146). Glutathione (GSH) levels in plasma and tissues were measured using a competitive‐ELISA kit (Elabscience, Cat. No: E‐EL‐0026). Additionally, catalase (CAT) and superoxide dismutase (SOD) activities in plasma and tissues were assessed using rat total SOD (FineTest, Cat No: ER1950) and CAT (USCN, Cat. No: SEC. 418Hu) ELISA kits, respectively. Protein concentrations in tissue samples were measured using a total protein colorimetric assay kit (Quick Start Bradford Protein Assay, Bio‐Rad).

### 2.5 mRNA Expression

Total RNA was isolated from kidney and liver tissue samples of rats using the RNA Purification Kit (Gene Jet, ThermoFisher Scientific, USA). To ensure the removal of any remaining DNA, the extracted RNA was handled with DNase I (RNase‐free, 1 U/μL, ThermoFisher Scientific, USA). Subsequently, the purified RNA was reverse‐transcribed into cDNA using the cDNA Reverse Transcription Kit (ThermoFisher Scientific, USA). Target genes were amplified using specific primers, as outlined in Table 1. Primers specific to *Rattus norvegicus* were designed by the authors for this study using the FastPCR 6.0 software [15], based on mRNA sequences for *β‐actin* (housekeeping gene), *TNF‐α*, *NFκB*, *Bcl‐2*, *Cas‐3*, and *Bax* obtained from the NCBI database. For RT‐qPCR, 1 μL of cDNA was combined with Master Mix Green (RealQ Plus 2x, Ampliqon, Denmark) and gene‐specific primers, and the reactions were performed on an RT‐qCR system (CFX96 Bio‐Rad, USA). The amplification specificity was verified by melt‐curve analysis, and data were collected using the manager software. All samples were checked against β‐actin, and results were stated as fold changes in cycle threshold (Ct) values relative to control samples, calculated using the 2^−ΔΔCt^ method [16].

**Table 1:** Oligonucleotide sequence, product size, and gene bank number of genes.

Gene	Oligonucleotide sequence		Product size (bp)	Gene bank number
*β‐Actin*	F	GAGGGAAATCGTGCGTGACAT	452	NC_005111.4
	R	ACATCTGCTGGAAGGTGGACA		
*Caspase‐3*	F	ACCCTGAAATGGGCTTGTGTA	427	NM_012922.2
	R	GCCATATCATCGTCAGTTCCAC		
*Bcl‐2*	F	GGGTATGATAACCGGGAGATCG	508	NM_016993.1
	R	ACTCAGTCATCCACAGAGCGA		
*NFκB*	F	TCCCCAAGCCAGCACCCCAGC	334	NM_199267.2
	R	GGCCCCCAAGTCTTCATCAGC		
*TNF‐α*	F	CGAGTGACAAGCCCGTAGCC	368	NM_012675.3
	R	GGATGAACACGCCAGTCGCC		
*Bax*	F	AGGACGCATCCACCAAGAAGC	363	NM_017059.2
	R	CAGTGAGGACTCCAGCCACAA		

### 2.6 Immunohistochemical Staining

Samples were initially processed with xylene and a graded alcohol series, and to inhibit endogenous peroxidase activity, an H_2_O_2_ (1%) solution was engaged for 30 min at 25°C. These samples were rinsed in distilled water for 2–3 min, immersed in citrate buffer, and heated for 10 min. Following washes with PBS and distilled water, the tissue sections were treated with a UV block for 10 min. Primary antibodies for *Bax* (1:100, sc7480, Santa Cruz), *Bcl‐2* (1:100, sc7382, Santa Cruz), and *TNF‐α* (1:100, PAA133Ra01; Cloud‐Clone Corp.) were then applied, and the slides were incubated for 12 h at 4°C. Slides washed using PBS solution were incubated with secondary antibody (100 µL, HRP Goat Anti‐Mouse IgG, Cat: PHL170627, Thermo Scientific, USA) for 10 min at room temperature. Detection was conducted using DAB substrate (Cat. No. 1855920; Thermo Scientific, USA), followed by 30 s of counterstaining with Gill's hematoxylin. The slides were scrutinized under a light microscope (Nikon, Tokyo, Japan) with ×200 magnification.

### 2.7 Histopathological Examination

Formaldehyde‐preserved tissue samples were processed for paraffin embedding, and thin sections (5μm) were obtained. These sections were stained using hematoxylin and eosin (H&E) according to the method outlined by Tureyen et al. [17]. The stained sections were identified using a light microscope (Nikon, Tokyo, Japan), and images were recorded with a camera (Nikon, Japan). Group differences were statistically analyzed based on tissue damage scores, categorized as follows: (0) no observed damage, (1) mild damage, (2) moderate damage, and (3) severe damage.

### 2.8 Statistical Analysis

The data from the study were statistically evaluated through GraphPad Prism 8.0 software. Initially, the normality of the data distribution was evaluated. For normally distributed data, a one‐way ANOVA was conducted, and Tukey's post hoc test was applied to determine variations among the groups. The results are presented as mean ± standard deviation, with statistical significance defined as *p* < 0.05.

## 3 Results

### 3.1 Taurine Ameliorates MTF‐Induced Liver and Kidney Function Parameters

**Figure 1:**
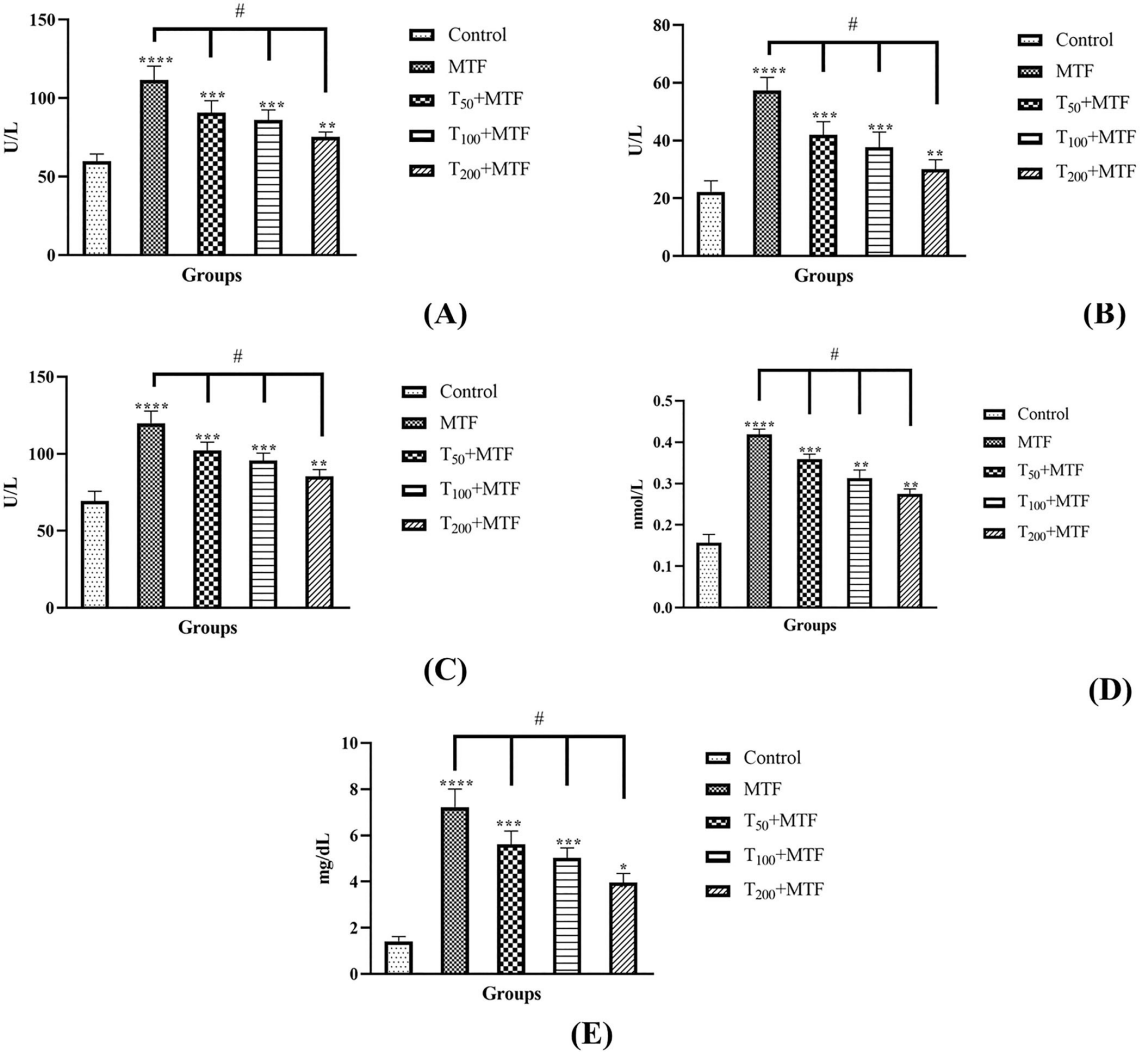
Effects of taurine (T) and metaflumizone (MTF) administration on AST (A), ALT (B), and ALP (C) activities and BUN (D) and creatinine (E) levels. Groups; Control, MTF (500 mg/kg), T_50_ + MTF (50 mg/kg T + 500 mg/kg MTF), T_100_ + MTF (100 mg/kg T + 500 mg/kg MTF), and T_200_ + MTF (200 mg/kg T + 500 mg/kg MTF). Mean ± standard deviations; *n*: 6. When the values shown with different stars (****p < 0.0001; ***p < 0.001; **p < 0.01; *p < 0.05) in the figure are compared with the control group and when the values shown with # are compared with the MTF group, it is statistically significant (*p* < 0.0001).

The activities of hepatic function markers, AST (Figure 1A), ALT (Figure 1B), and ALP (Figure 1C), along with kidney function parameters, creatinine (Figure 1D), and BUN (Figure 1D), were observed to be significantly elevated among the group treated with MTF relative to the control group (*p* < 0.0001). Nevertheless, taurine applications in a dose‐dependent manner notably mitigated the increase induced by MTF (*p* < 0.0001).

### 3.2 Taurine Improved MTF‐Induced MDH and Antioxidant Enzyme Activities

**Figure 2:**
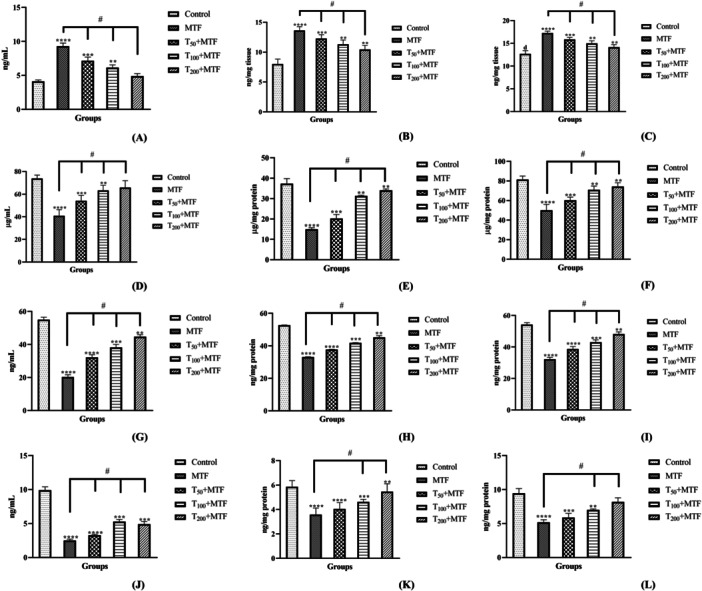
Effects of taurine (T) and metaflumizone (MTF) administration on plasma, hepatic and renal MDH (A, B, and C) and GSH (D, E, and F) levels and SOD (G, H, and I) and CAT (J, K, and L) activities. Groups; Control, MTF (500 mg/kg), T_50_ + MTF (50 mg/kg T + 500 mg/kg MTF), T_100_ + MTF (100 mg/kg T + 500 mg/kg MTF), and T_200_ + MTF (200 mg/kg T + 500 mg/kg MTF). Mean ± standard deviations; *n*: 6. When the values shown with different stars (****p < 0.0001; ***p < 0.001; **p < 0.01) in the figure are compared with the control group and when the values shown with # are compared with the MTF group, it is statistically significant (*p* < 0.0001).

MTF administration caused a marked rise in MDH levels in the plasma (Figure 2A), liver (Figure 2B), and kidney (Figure 2C) of rats relative to the control group, whereas taurine treatment progressively lowered these levels in a dose‐dependent manner (*p *< 0.0001). Also, MTF administration notably decreased the GSH levels in plasma (Figure 2D), liver (Figure 2E), and kidney (Figure 2F) (*p *< 0.0001). The activities of SOD in plasma (Figure 2G), liver (Figure 2H), and kidney (Figure 2I), as well as CAT in plasma (Figure 2J), liver (Figure 2K), and kidney (Figure 2L) were also decreased by MTF treatment (*p* < 0.0001). Nevertheless, the taurine treatments in a dose‐dependent manner effectively counteracted the MTF‐induced alterations, restoring the values to levels comparable to the control group (*p* < 0.0001).

### 3.3 Taurine Ameliorates mRNA and Protein Expression Increased by MTF

**Figure 3:**
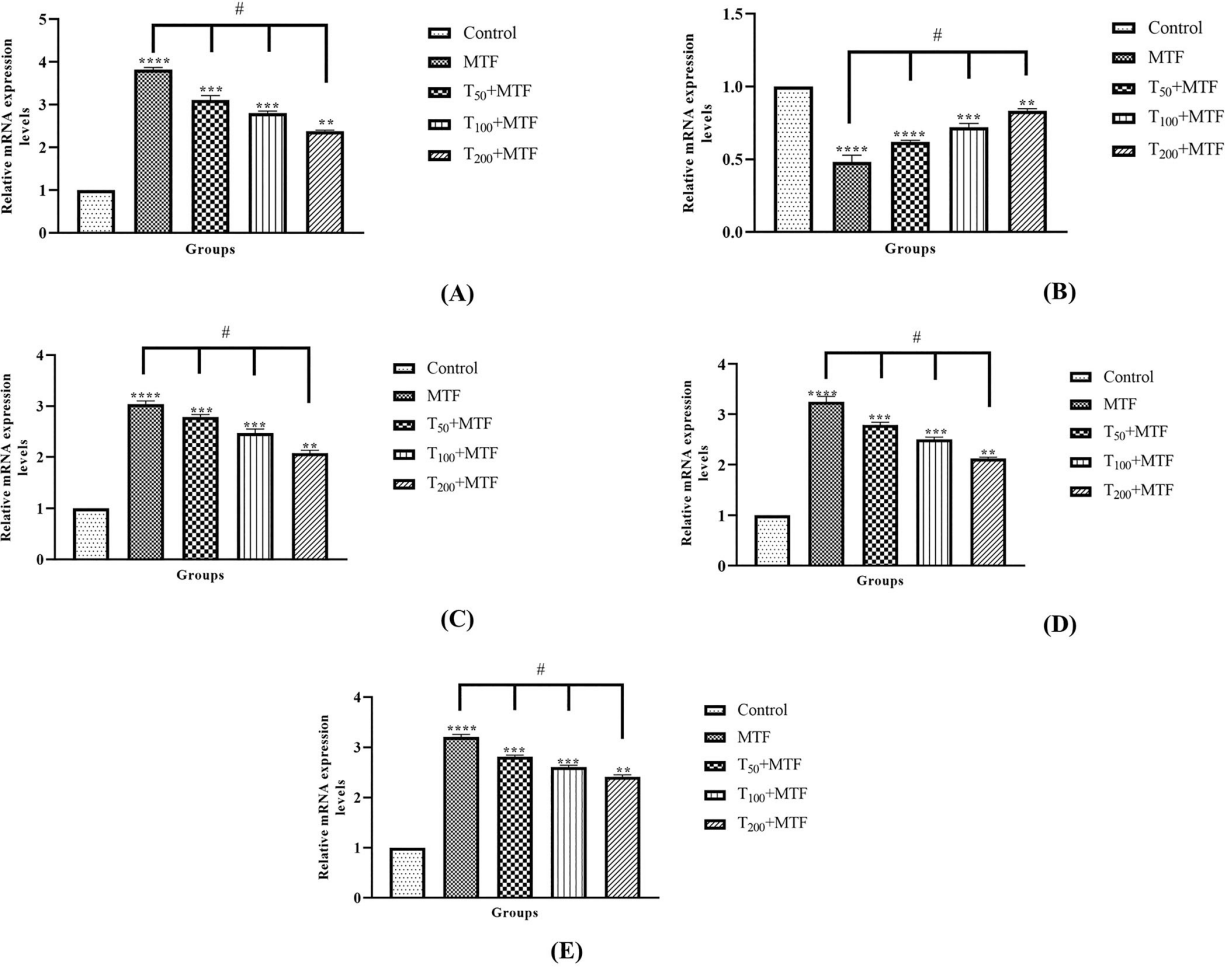
Effects of taurine (T) and metaflumizone (MTF) administration on expression levels of *Bax* (A), *Bcl‐2* (B), *Cas‐3* (C), *NFκB* (D), and *TNF‐α* (E), which play a role in the apoptotic and inflammatory process in liver tissue. Groups; Control, MTF (500 mg/kg), T_50_ + MTF (50 mg/kg T + 500 mg/kg MTF), T_100_ + MTF (100 mg/kg T + 500 mg/kg MTF), and T_200_ + MTF (200 mg/kg T + 500 mg/kg MTF). Mean ± standard deviations; *n*: 6. When the values shown with different stars (****p < 0.0001; ***p < 0.001; **p < 0.01) in the figure are compared with the control group and when the values shown with # are compared with the MTF group, it is statistically significant (*p* < 0.0001).

**Figure 4:**
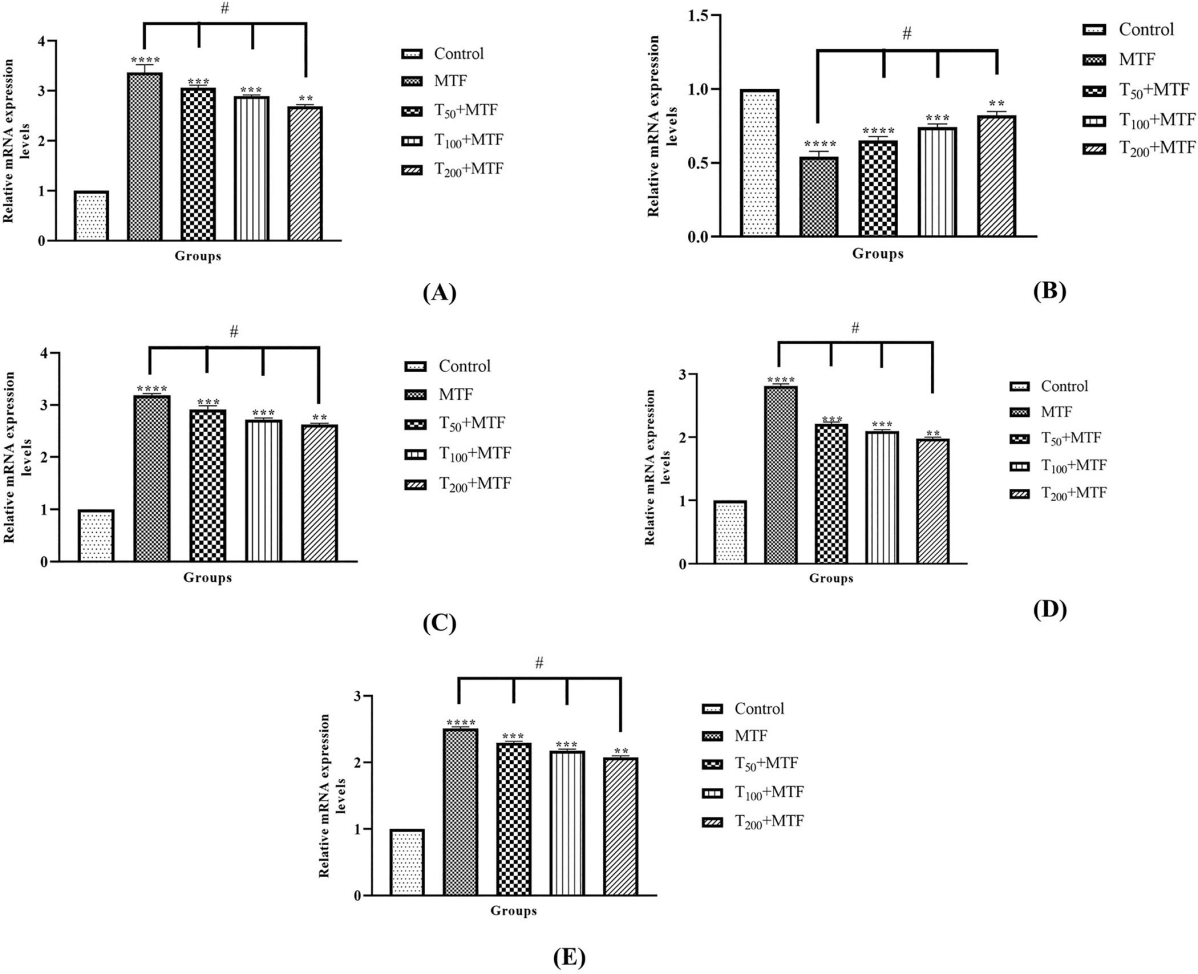
Effects of taurine (T) and metaflumizone (MTF) administration on expression levels of *Bax* (A), *Bcl‐2* (B), *Cas‐3* (C), *NFκB* (D), and *TNF‐α* (E), which play a role in the apoptotic and inflammatory process in kidney tissue. Groups; Control, MTF (500 mg/kg), T_50_ + MTF (50 mg/kg T + 500 mg/kg MTF), T_100_ + MTF (100 mg/kg T + 500 mg/kg MTF), and T_200_ + MTF (200 mg/kg T + 500 mg/kg MTF). Mean ± standard deviations; *n*: 6. When the values shown with different stars (****p < 0.0001; ***p < 0.001; **p < 0.01) in the figure are compared with the control group and when the values shown with # are compared with the MTF group, it is statistically significant (*p* < 0.0001).

**Figure 5:**
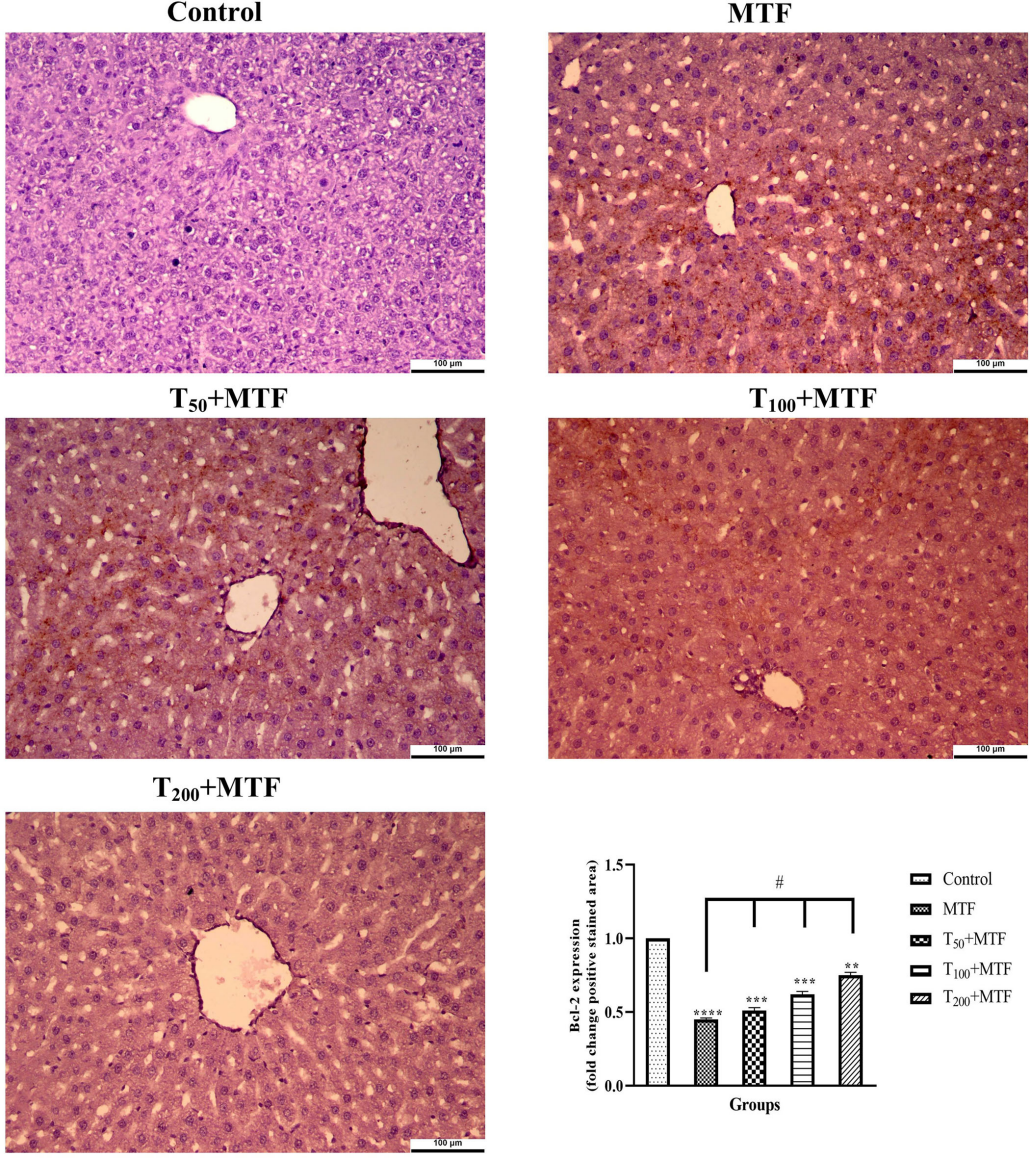
*Bcl‐2* immunohistochemical staining and analyses in liver tissue following taurine (T) and metaflumizone (MTF) applications. There are many positive cells (brown stain) in MTF groups, but they markedly decreased in the T groups in a dose‐dependent manner. Groups; Control, MTF (500 mg/kg), T_50_ + MTF (50 mg/kg T + 500 mg/kg MTF), T_100_ + MTF (100 mg/kg T + 500 mg/kg MTF), and T_200_ + MTF (200 mg/kg T + 500 mg/kg MTF). Mean ± standard deviations; *n*: 6. When the values shown with different stars (****p < 0.0001; ***p < 0.001; **p < 0.01) in the figure are compared with the control group and when the values shown with # are compared with the MTF group, it is statistically significant (*p* < 0.0001).

**Figure 6:**
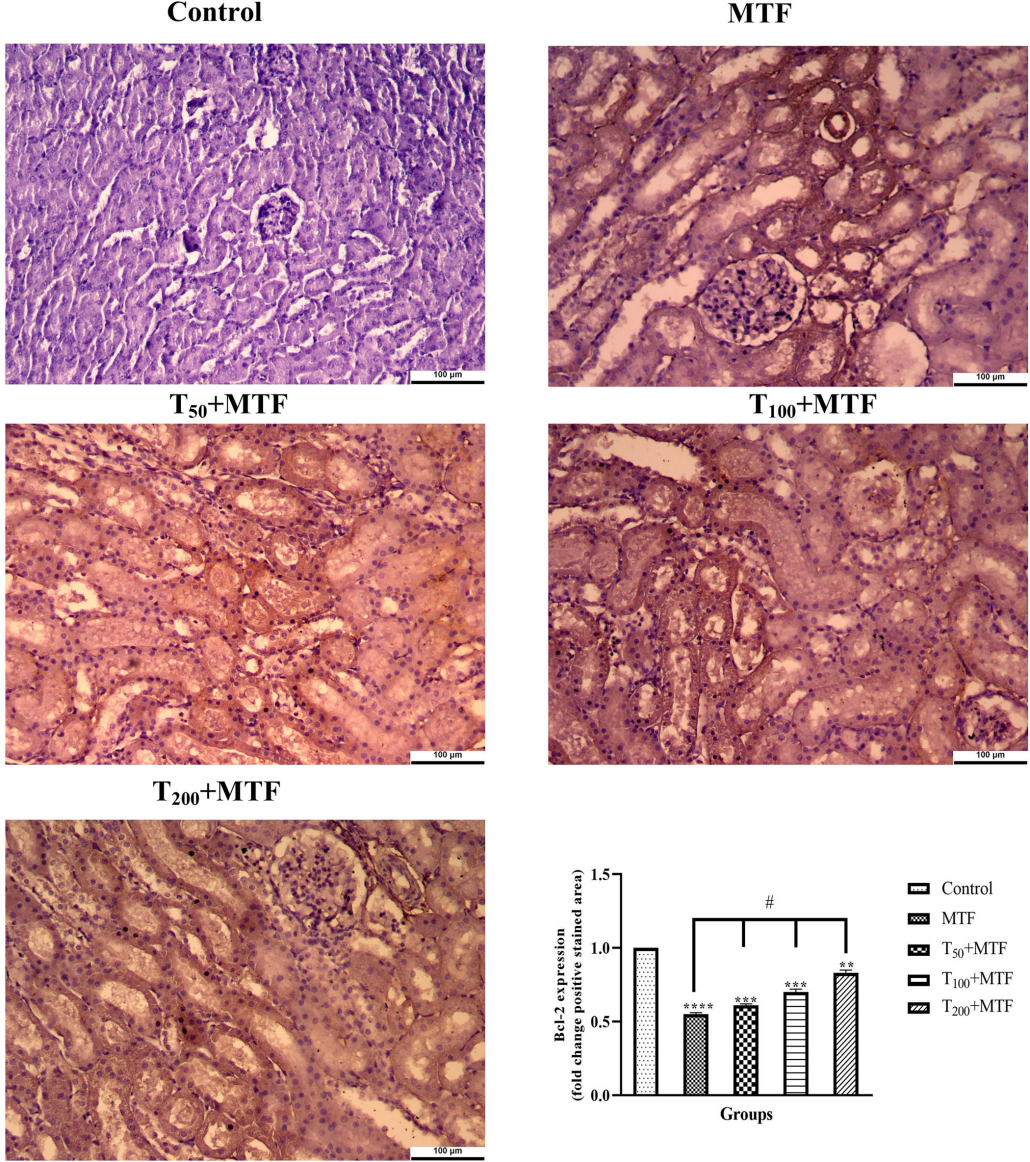
*Bcl‐2* immunohistochemical staining and analyses in kidney tissue following taurine (T) and metaflumizone (MTF) applications. There are many positive cells (brown stain) in MTF groups, but they markedly decreased in the T groups in a dose‐dependent manner. Groups; Control, MTF (500 mg/kg), T_50_ + MTF (50 mg/kg T + 500 mg/kg MTF), T_100_ + MTF (100 mg/kg T + 500 mg/kg MTF), and T_200_ + MTF (200 mg/kg T + 500 mg/kg MTF). Mean ± standard deviations; *n*: 6. When the values shown with different stars (****p < 0.0001; ***p < 0.001; **p < 0.01) in the figure are compared with the control group and when the values shown with # are compared with the MTF group, it is statistically significant (*p* < 0.0001).

**Figure 7:**
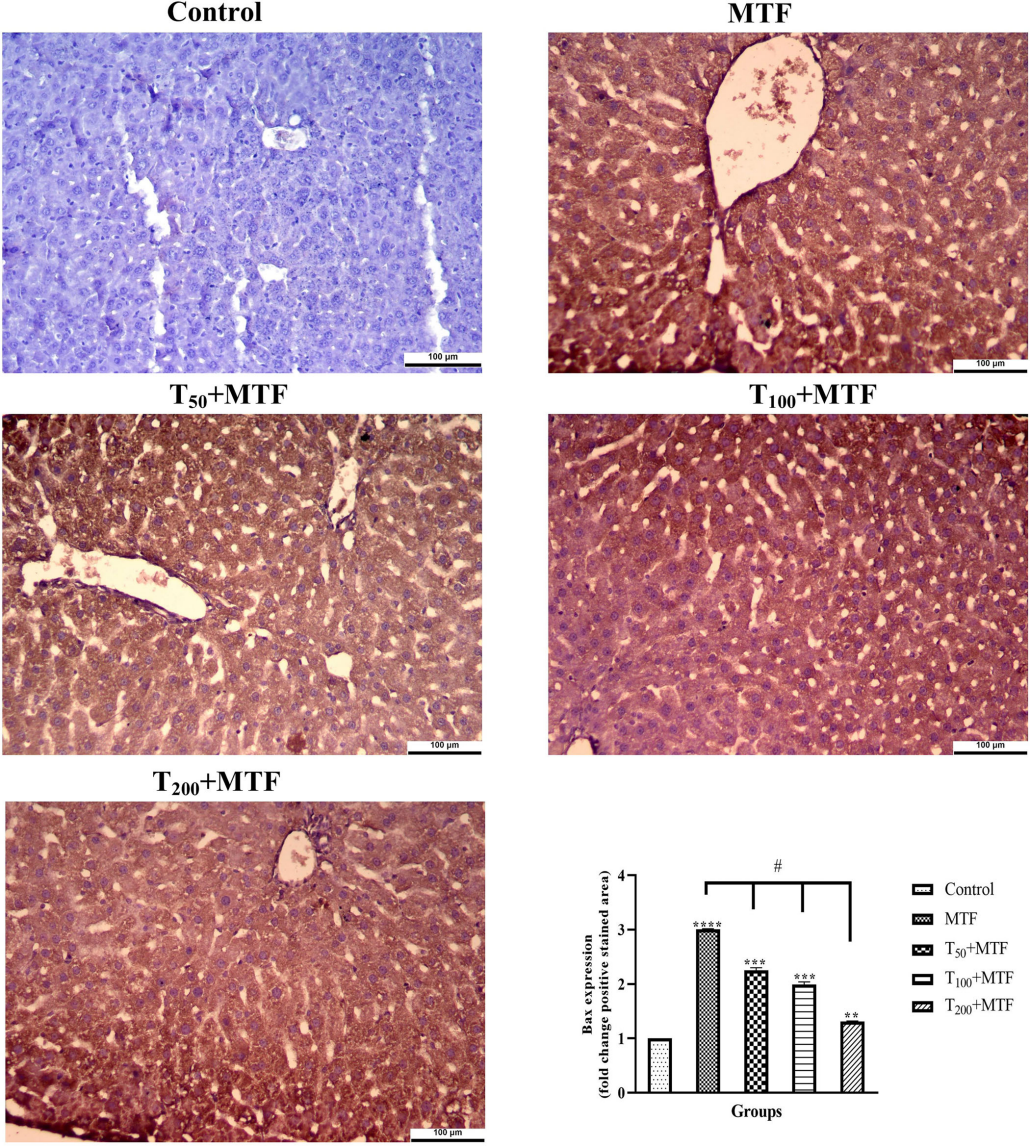
*Bax* immunohistochemical staining and analyses in liver tissue following taurine (T) and metaflumizone (MTF) applications. There are many positive cells (brown stain) in MTF groups, but they markedly decreased in the T groups in a dose‐dependent manner. Groups; Control, MTF (500 mg/kg), T_50_ + MTF (50 mg/kg T + 500 mg/kg MTF), T_100_ + MTF (100 mg/kg T + 500 mg/kg MTF), and T_200_ + MTF (200 mg/kg T + 500 mg/kg MTF). Mean ± standard deviations; *n*: 6. When the values shown with different stars (****p < 0.0001; ***p < 0.001; **p < 0.01) in the figure are compared with the control group and when the values shown with # are compared with the MTF group, it is statistically significant (*p* < 0.0001).

**Figure 8:**
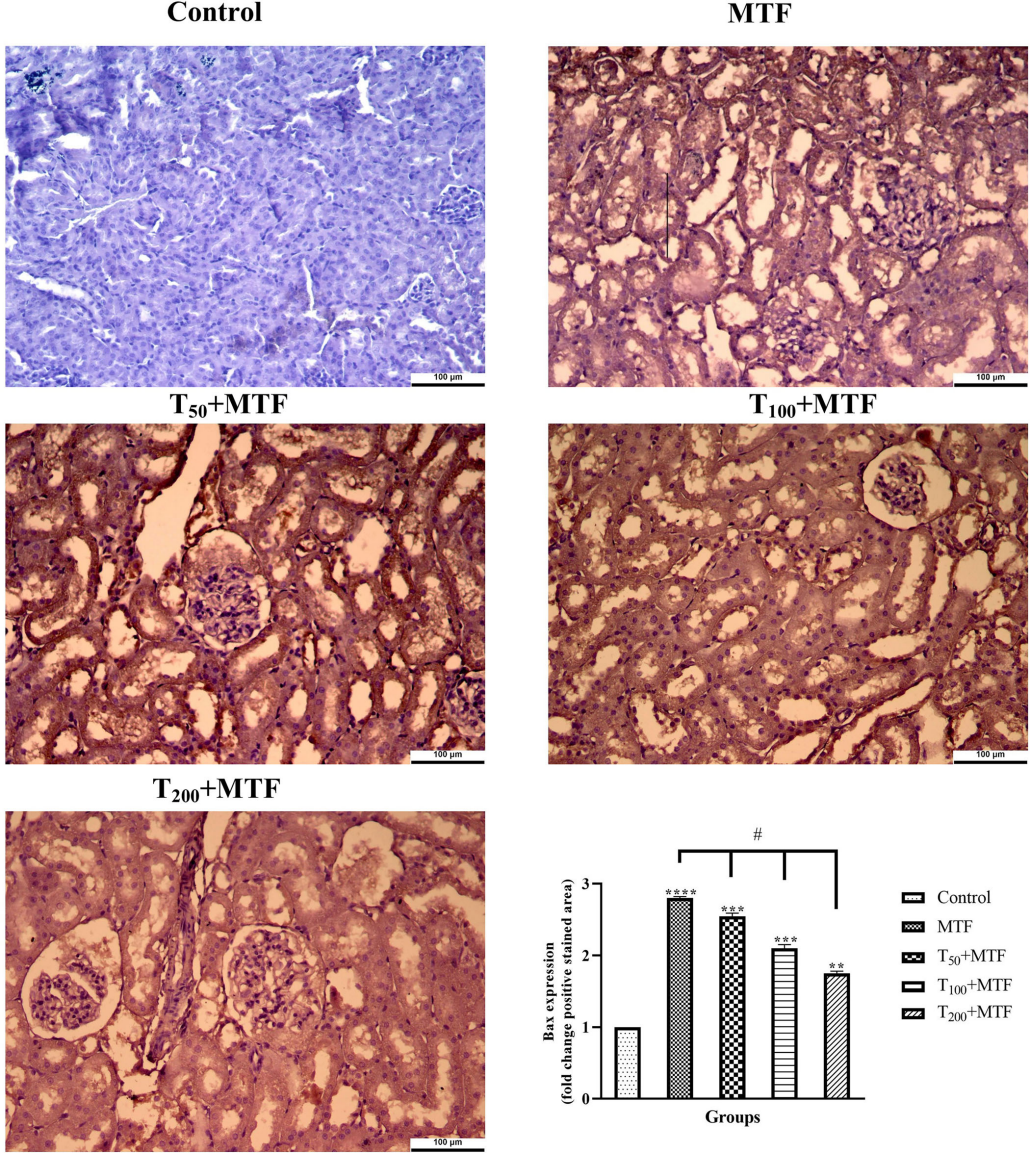
*Bax* immunohistochemical staining and analyses in kidney tissue following taurine (T) and metaflumizone (MTF) applications. There are many positive cells (brown stain) in MTF groups, but they markedly decreased in the T groups in a dose‐dependent manner. Groups; Control, MTF (500 mg/kg), T_50_ + MTF (50 mg/kg T + 500 mg/kg MTF), T_100_ + MTF (100 mg/kg T + 500 mg/kg MTF), and T_200_ + MTF (200 mg/kg T + 500 mg/kg MTF). Mean ± standard deviations; *n*: 6. When the values shown with different stars (****p < 0.0001; ***p < 0.001; **p < 0.01) in the figure are compared with the control group and when the values shown with # are compared with the MTF group, it is statistically significant (*p* < 0.0001).

**Figure 9:**
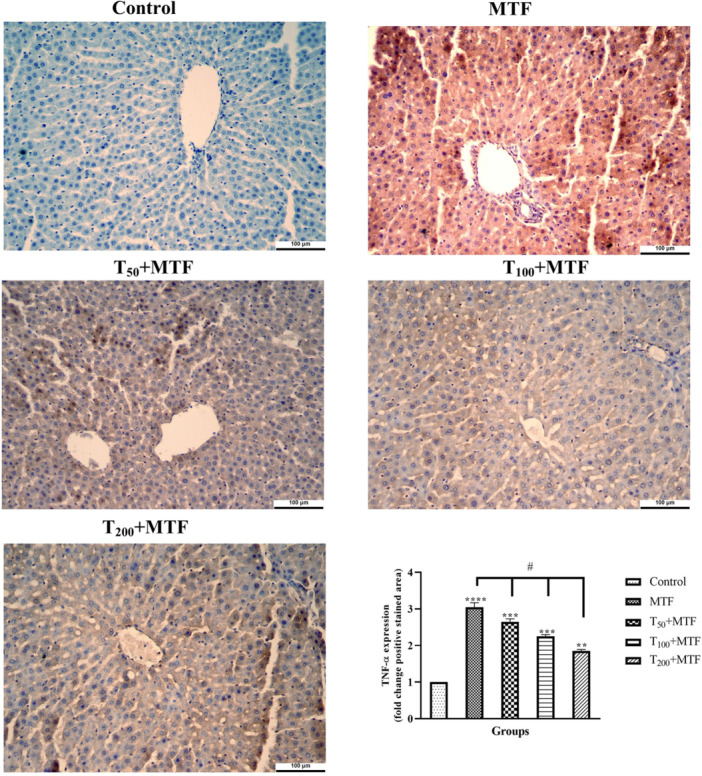
*TNF‐α* immunohistochemical staining and analyses in liver tissue following taurine (T) and metaflumizone (MTF) applications. There are many positive cells (brown stain) in MTF groups, but they markedly decreased in the T groups in a dose‐dependent manner. Groups; Control, MTF (500 mg/kg), T_50_ + MTF (50 mg/kg T + 500 mg/kg MTF), T_100_ + MTF (100 mg/kg T + 500 mg/kg MTF), and T_200_ + MTF (200 mg/kg T + 500 mg/kg MTF). Mean ± standard deviations; *n*: 6. When the values shown with different stars (****p < 0.0001; ***p < 0.001; **p < 0.01) in the figure are compared with the control group and when the values shown with # are compared with the MTF group, it is statistically significant (*p* < 0.0001).

**Figure 10:**
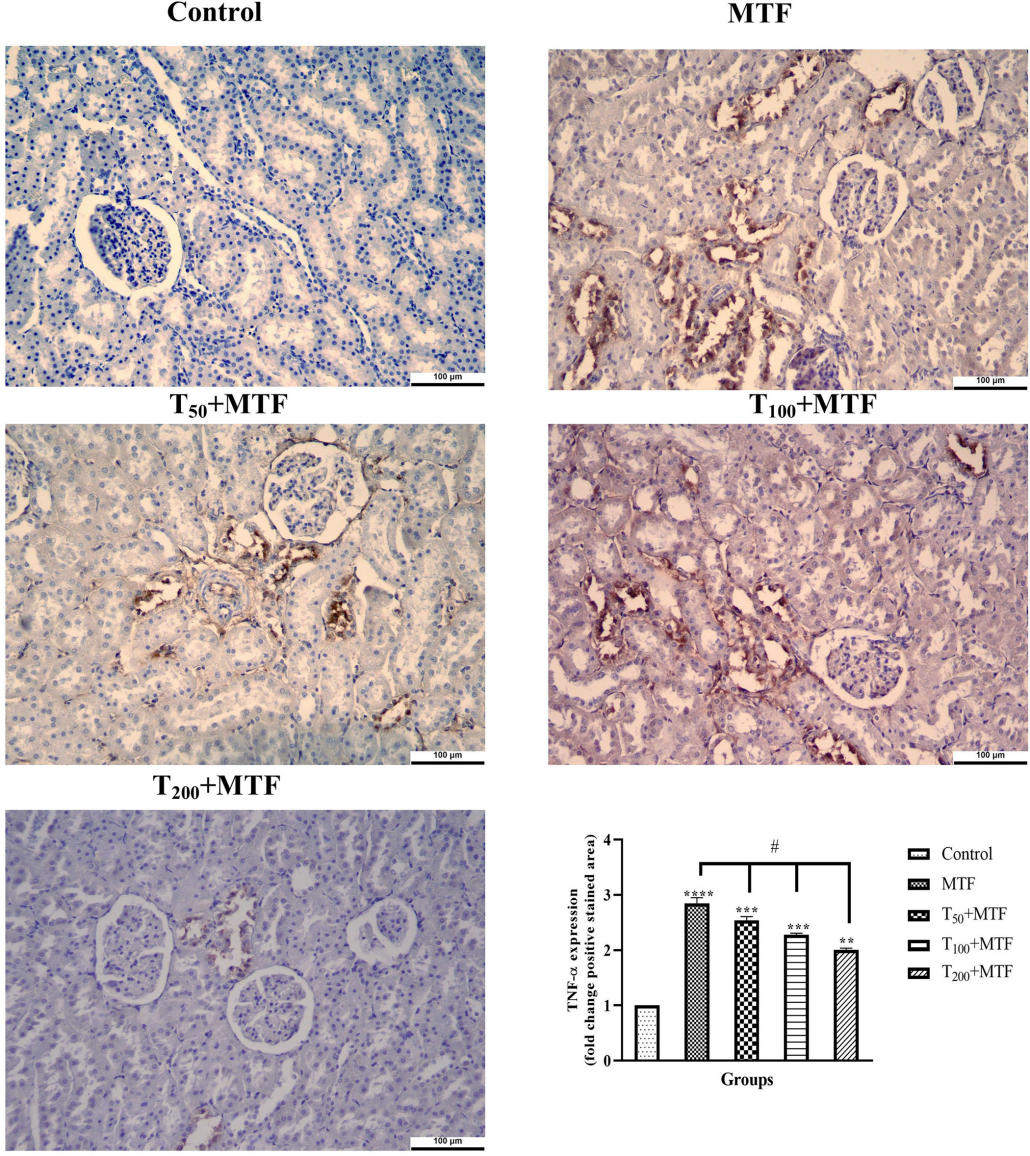
*TNF‐α* immunohistochemical staining and analyses in kidney tissue following taurine (T) and metaflumizone (MTF) applications. There are many positive cells (brown stain) in MTF groups, but they markedly decreased in the T groups in a dose‐dependent manner. Groups; Control, MTF (500 mg/kg), T_50_ + MTF (50 mg/kg T + 500 mg/kg MTF), T_100_ + MTF (100 mg/kg T + 500 mg/kg MTF), and T_200_ + MTF (200 mg/kg T + 500 mg/kg MTF). Mean ± standard deviations; *n*: 6. When the values shown with different stars (****p < 0.0001; ***p < 0.001; **p < 0.01) in the figure are compared with the control group and when the values shown with # are compared with the MTF group, it is statistically significant (*p* < 0.0001).

MTF administration decreased mRNA expression level of *Bcl‐2* (Figure 3B) but enhanced *Bax* (Figure 3A), *Cas‐3* (Figure 3C), *TNF‐α* (Figure 3D), and *NFκB* (Figure 3E) levels in the liver (Figure 2B) compared to the control group (*p *< 0.0001). Similarly, in the kidney (Figure 2C) of rats, MTF administration decreased mRNA expression level of *Bcl‐2* (Figure 4B) but enhanced *Bax* (Figure 4A), *Cas‐3* (Figure 4C), *TNF‐α* (Figure 4D), and *NFκB* (Figure 4E) levels relative to the control group (*p* < 0.0001). Unlike MTF administration, taurine treatments in a dose‐dependent manner progressively regulated these levels (*p *< 0.0001). These changes were further supported by decreased *Bcl‐2* protein expression in the liver (Figure 5) and kidney (Figure 6), while increased *Bax* in the liver (Figure 7) and kidney (Figure 8) (*p* < 0.0001). Additionally, MTF treatment increased *TNF‐α* protein levels in the liver (Figure 9) and kidney (Figure 10) tissues (*p* < 0.0001). Taurine treatments in a dose‐dependent manner; nevertheless, modulated these MTF‐induced alterations, bringing both mRNA and protein expression levels approaching the levels observed in the control group (*p* < 0.0001).

### 3.4 Taurine Improved MTF‐Induced Liver and Kidney Tissue Damage

**Figure 11:**
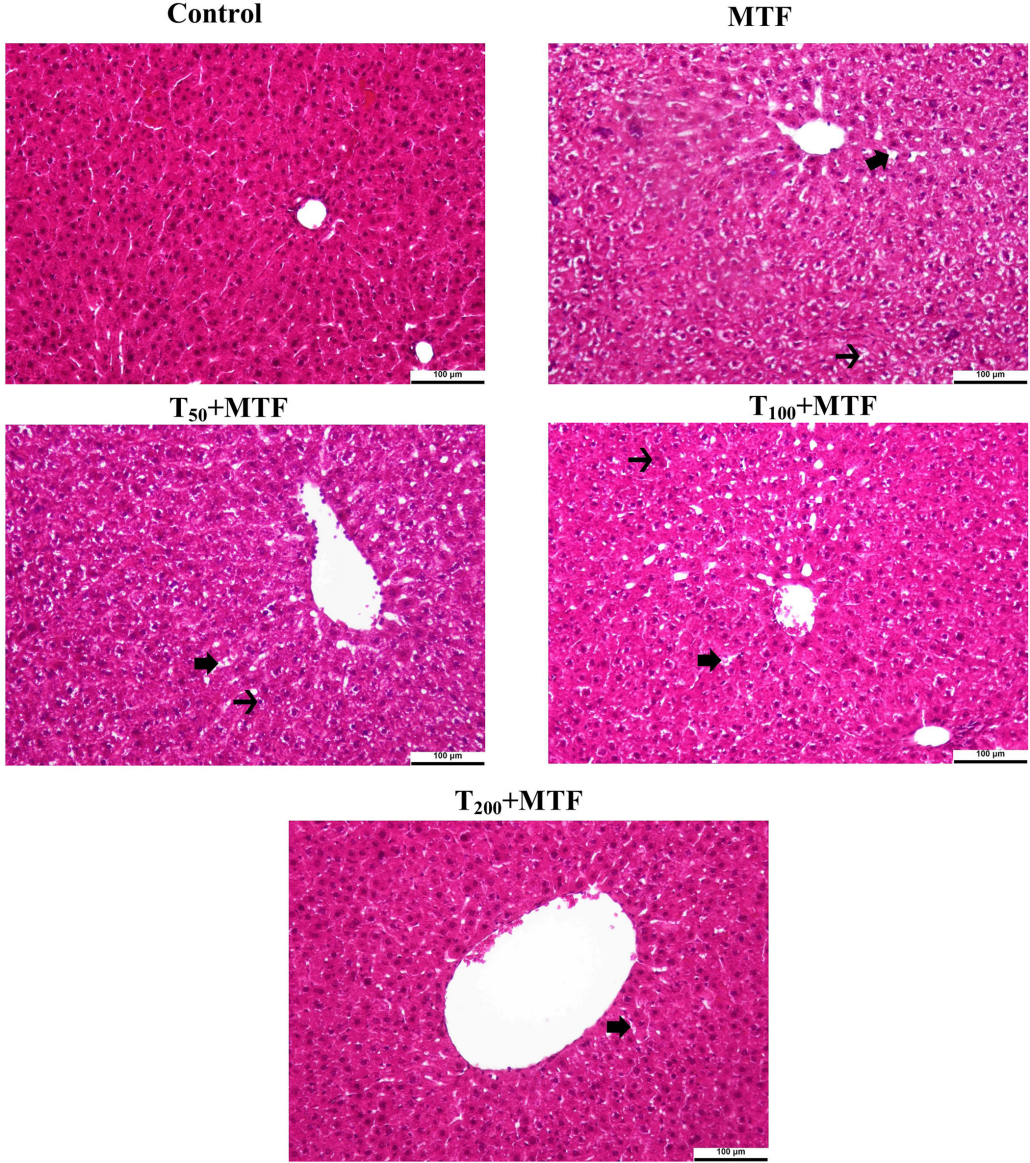
Histopathological changes in liver tissue of rats induced by taurine (T) and metaflumizone (MTF) treatments. All figures are H&E stained. Original magnification is 20× and 100 µm. Degenerative changes in hepatocytes (thin arrow), sinusoidal dilatation, and hyperemia (thick arrow). Control, MTF (500 mg/kg), T_50_ + MTF (50 mg/kg T + 500 mg/kg MTF), T_100_ + MTF (100 mg/kg T + 500 mg/kg MTF), and T_200_ + MTF (200 mg/kg T + 500 mg/kg MTF).

**Figure 12:**
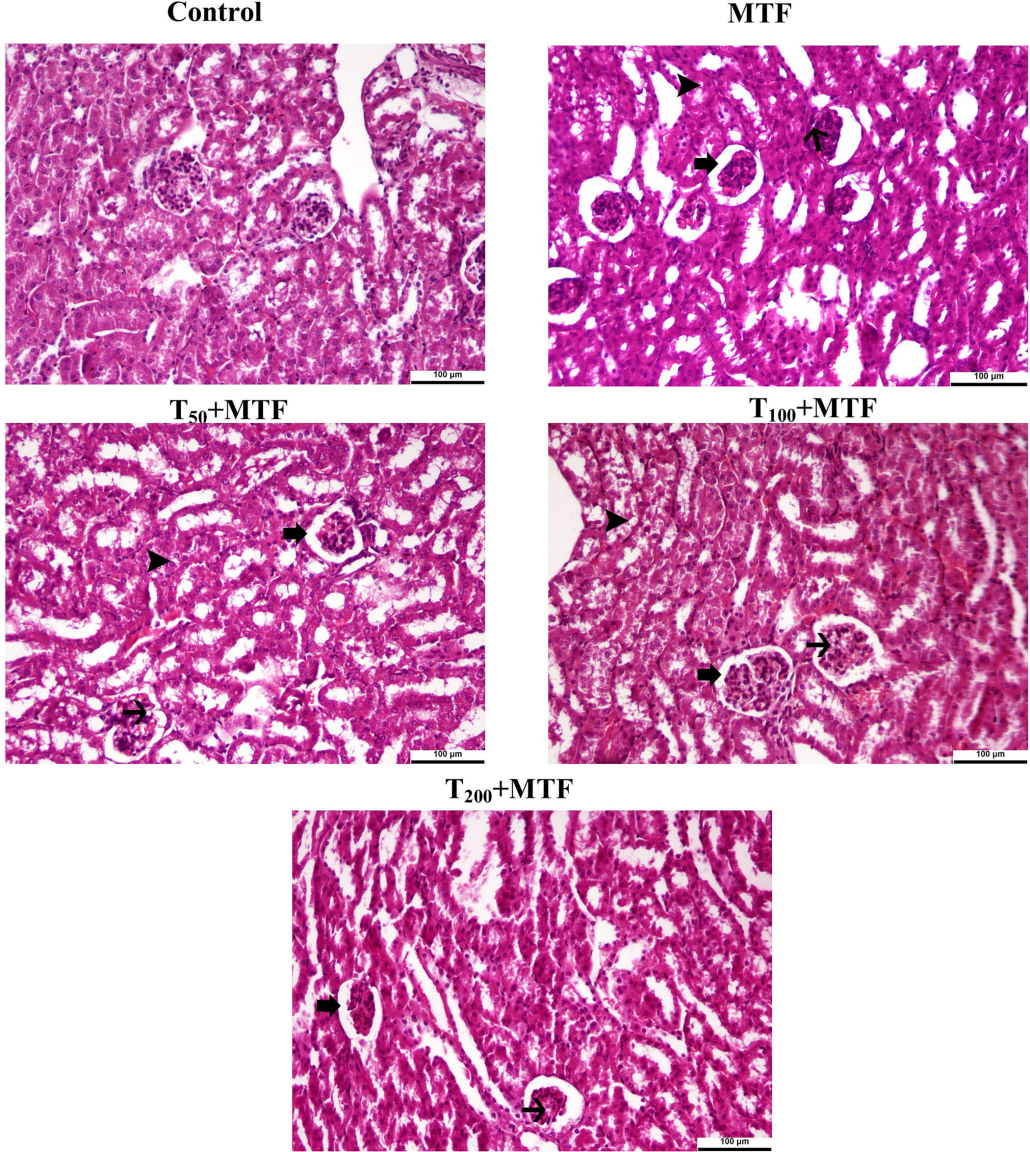
Histopathological changes in kidney tissue of rats after taurine (T) and metaflumizone (MTF) treatments. All figures are stained with H&E. Original magnification is 20× and 100 µm. Enlargement of glomerular Bowman space (thick arrow), vacuolization formations in glomerulus capillaries (thin arrow), and areas of vacuolar degeneration in tubules (arrowhead). Control, MTF (500 mg/kg), T_50_ + MTF (50 mg/kg T + 500 mg/kg MTF), T_100_ + MTF (100 mg/kg T + 500 mg/kg MTF), and T_200_ + MTF (200 mg/kg T + 500 mg/kg MTF).

MTF treatment resulted in degenerative changes in hepatocytes, hyperemia, and sinusoidal dilatation in the liver tissue (Figure 11). The kidney tissue, MTF administration caused enlargement of the glomerular Bowman space, areas of vacuolar degeneration in the glomerular capillary bundle, and vacuolization formations in glomerulus capillaries (Figure 12). However, taurine treatments in a dose‐dependent manner were observed to reduce these MTF‐induced damages in both the liver and kidney tissues. Moreover, the histopathological statistical analyses of the kidney and liver were expressed in Table 2.

**Table 2:** Effect of the metaflumizone and 50, 100, and 200 mg/kg taurine administration for 30 days on histopathological changes in liver and kidney tissues stained with H&E.

Tissue	Histopathological alterations	Control	MTF	T_50_ + MTF	T_100_ + MTF	T_200_ + MTF
Liver	Sinusoidal dilatation and hyperemia	0.00 ± 0.00^e^	3.01 ± 0.01^a^	2.00 ± 0.01^b^	1.54 ± 0.51^c^	1.01 ± 0.01^d^
	Degenerative changes in hepatocytes	0.00 ± 0.00^e^	2.83 ± 0.41^a^	2.01 ± 0.01^b^	1.51 ± 0.54^c^	1.01 ± 0.01^d^
Kidney	Glomerulus in Bowman's cavity expansion	0.00 ± 0.00^c^	2.51 ± 0.54^a^	2.16 ± 0.41^a^	1.51 ± 0.54^b^	1.01 ± 0.01^b^
	Vacuolization formations in glomerulus capillaries	0.00 ± 0.00^d^	2.33 ± 0.51^a^	2.01 ± 0.01^ab^	1.83 ± 0.01^b^	1.01 ± 0.01^c^
	Areas of vacuolar degeneration in tubules	0.00 ± 0.00^e^	2.51 ± 0.54^a^	2.33 ± 0.51^b^	1.83 ± 0.41^c^	1.01 ± 0.01^d^

*Note:* Groups: Control, MTF (500 mg/kg), T_50_ + MTF (50 mg/kg T + 500 mg/kg MTF), T_100_ + MTF (100 mg/kg T + 500 mg/kg MTF), and T_200_ + MTF (200 mg/kg T + 500 mg/kg MTF). Mean±standart deviation; *n*: 6. When compared with the control group, the data shown with letters a, b, c, d, e were found to be statistically different (*p* < 0.05).

Abbreviations: MTF, metaflumizone; T, Taurin.

## 4 Discussion

This study presented the protective role of taurine in preventing MTF‐induced hepatorenal injury caused by oxidative stress, inflammation, and apoptosis, supported by biochemical, molecular, and histopathological analyses.

SBIs are considered safer for mammals; their toxicity to the liver and kidneys may depend on the dose and duration of exposure [18, 19, 20]. Indeed, administration of indoxacarb at doses of 15, 30, and 60 mg/kg for 60 days in rats has been documented to enhance serum BUN, AST, ALT, and creatinine levels [18]. Similarly, exposure of roosters to 250 ppm indoxacarb was found to elevate BUN, creatinine, ALT, AST, and ALP levels. Furthermore, administration of indoxacarb at 50%, 25%, and 10% of its lethal dose to chickens resulted in increased ALP, SGPT, AST, and creatinine levels [21]. Moreover, Serim et al. [13] demonstrated that taurine mitigated the elevated biochemical parameters (BUN, AST, ALT, ALP, and creatinine) induced by pyraclostrobin toxicity and provided tissue protection against damage. Consistently, the present study also revealed that taurine alleviated MTF‐induced increases in biochemical parameters, suggesting a safeguarding impact on hepatic and renal tissues.

SBI exposure causes deterioration of the activities of mitochondrial enzymes such as MDH due to increased oxidative stress [19]. This disruption leads to the shutdown of mitochondrial oxidative phosphorylation and the tricarboxylic acid cycle, ultimately inhibiting mitochondrial ATP production [22]. As highlighted in recent studies [19, 23, 24] SBI‐induced oxidative stress has been demonstrated through increased lipid peroxidation, decreased GSH levels, and reduced functions of antioxidative enzymes like SOD and CAT in the kidney and livers of SBI‐exposed rodents. In contrast, the present study revealed that taurine at three different doses mitigated these MTF‐induced alterations in tissue parameters. Consistent with previous findings, this suggests that taurine exerts an inhibitory effect on oxidative stress by enhancing antioxidant defense mechanisms [13, 25].

Activation of the inflammatory and apoptotic pathways is also implicated in SBI‐mediated toxicity [26]. Tissue injury may be facilitated by the activation of cells, which upregulate inflammatory cytokines such as *TNF‐α*, *IL‐1β*, and *IL‐6*, which may increase toxicity [27]. Similarly, MTF increased *TNF‐α* and *NFκB* expression was observed in both the kidney and liver. Correlations between insecticide exposure and cytokine activity alterations have been previously reported [28]. Gargouri et al. [29] demonstrated that bifenthrin, a sodium channel‐blocking insecticide, significantly increased *NFκB* protein and mRNA levels in human neuroblastoma cells, leading to the transcription of pro‐inflammatory cytokines such as *TNF‐α* and inducing oxidative stress‐related markers. Additionally, various insecticides have been suggested to trigger reactive oxygen species production, thereby exacerbating oxidative stress and activating the *NFκB* signaling pathway [30]. Hassanen et al. [28] reported that increased lipid peroxidation levels in the kidneys and livers of rats exposed to imidacloprid and hexaflumuron contributed to toxicity via *NFκB* pathway activation and pro‐inflammatory cytokine induction. Furthermore, these rats exhibited elevated transcript levels of *Cas‐3*, *JNK*, and *HO‐1* genes, along with strong immunopositivity in *Cas‐3*, *TNF‐α*, and *NFκB* protein expressions. Moreover, natural pyrethrins are known to induce caspase‐driven apoptosis in hepatocytes via the mitochondrial pathway regulated by *Bax* and *Bcl‐2* [31]. Likewise, in the present study, MTF‐treated liver and kidney tissues exhibited high immunopositivity for *Bax* and *TNF‐α*, whereas *Bcl‐2* showed low immunopositivity. This finding suggests that MTF exerts a significant impact on the kidneys and livers by inducing pronounced oxidative stress damage, leading to mitochondrial dysfunction, *TNF‐α* and *NFκB‐*mediated inflammation, and *Cas‐3* and *Bax*‐mediated apoptosis.

Taurine has been reported to terminate glutamate‐induced toxicity by blocking increased intracellular calcium [32, 33]. This calcium increase is generally responsible for the activation of apoptotic cascades [34]. Apoptosis is also activated by neuroinflammatory pathways such as the *NF‐κB* system, and since taurine has an important effect in preventing this apoptosis [34, 35], whether it has any effect on SBI‐induced toxicity was evaluated in the study. In this study, taurine treatment suppressed *TNF‐α*, *NFκB*, *Bax*, and *Cas‐3* expression while increasing *Bcl‐2* levels in MTF‐treated rats, indicating its antiapoptotic and anti‐inflammatory characteristics. Consistent with this, previous studies have demonstrated that taurine exerts anti‐inflammatory and antiapoptotic effects by reducing *TNF‐α* expression in lipopolysaccharide‐induced liver injury [36], downregulating *Cas‐3* and *Cas‐9* expressions in γ‐irradiation‐induced liver damage in rats [37], and decreasing *TNF‐α* and *NFκB* expressions in thioacetamide‐induced kidney injury in rats [38].

Insecticide applications have been shown to cause damage to kidney and liver tissues [39]. Mossa et al. [40] documented degeneration, inflammatory cell infiltration, cellular proliferation, and focal hepatic hemorrhage in the livers of rats exposed to fipronil, as well as necrosis, glomerular tuft atrophy, vacuolation, and focal hemorrhage in the kidneys. Similarly, Koli et al. [18] observed severe hepatocyte swelling and degeneration, large fatty vacuoles in the cytoplasm of the liver, and a narrowed Bowman's capsule in the renal tubules of indoxacarb‐treated rats. Consistently, in this study, MTF administration led to structural alterations in the liver, including degenerative alterations in hepatocytes, sinusoidal dilatation, and hyperemia, as well as, in the kidney, it caused enlargement of the glomerular Bowman space, vacuolar degeneration in the glomerular capillary bundle, and vacuolization in glomerular capillaries. However, taurine treatment mitigated these histopathological changes in kidney and liver tissues, demonstrating its protective effects [13, 41].

## 5 Conclusion

Our study demonstrated that taurine administrations in a dose‐dependent manner prevented MTF‐induced oxidative stress, inflammation, and apoptosis in the kidney and liver of rats. The results suggest that taurine has potential as a candidate for the development of novel adjuvants or therapeutic agents aimed at counteracting the adverse effects of MTF on liver and kidney function. However, despite its promising preclinical protective effects, the study is limited by the lack of evaluation of taurine's clinical therapeutic efficacy. Future research should address these aspects to fully explore taurine's potential applications.

## Author Contributions

**Hasan Huseyin Demirel:** supervision, visualization, writing‐original draft, writing – review and editing, methodology, and conceptualization. **Sinan Ince:** data curation, investigation, methodology, writing – review and editing. **Fahriye Zemheri‐Navruz:** investigation and methodology. **Sevim Feyza Erdogmus:** investigation and methodology. **Nilay Isitez:** investigation and methodology.

## Ethics Statement

This study was conducted with the approval of the local ethics committee for animal experiments, Afyon Kocatepe University, Afyonkarahisar, Turkey (Approval Number: 49533702/20).

## Data Availability

All data generated or analyzed during this study are included in the manuscript.
